# A Unified Preprocessing Pipeline for Noise-Resilient Crack Segmentation in Leaky Infrastructure Surfaces

**DOI:** 10.3390/s25175574

**Published:** 2025-09-06

**Authors:** Jae-Jun Shin, Jeongho Cho

**Affiliations:** Department of Electrical Engineering, Soonchunhyang University, Asan 31538, Republic of Korea; grandfinal12@naver.com

**Keywords:** crack segmentation, deep learning, image preprocessing, infrastructure inspection, wet cracks

## Abstract

Wet cracks caused by leakage often exhibit visual and structural distortions due to surface contamination, salt crystallization, and corrosion byproducts. These factors significantly degrade the performance of sensor- and vision-based crack detection systems. In moist environments, the initiation and propagation of cracks tend to be highly nonlinear and irregular, making it challenging to distinguish crack regions from the background—especially under visual noise such as reflections, stains, and low contrast. To address these challenges, this study proposes a segmentation framework that integrates a dedicated preprocessing pipeline aimed at suppressing noise and enhancing feature clarity, all without altering the underlying segmentation architecture. The pipeline begins with adaptive thresholding to perform initial binarization under varying lighting conditions. This is followed by morphological operations and connected component analysis to eliminate micro-level noise and restore structural continuity of crack patterns. Subsequently, both local and global contrast are enhanced using histogram stretching and contrast limited adaptive histogram equalization. Finally, a background fusion step is applied to emphasize crack features while preserving the original surface texture. Experimental results demonstrate that the proposed method significantly improves segmentation performance under adverse conditions. Notably, it achieves a precision of 97.5% and exhibits strong robustness against noise introduced by moisture, reflections, and surface irregularities. These findings confirm that targeted preprocessing can substantially enhance the accuracy and reliability of crack detection systems deployed in real-world infrastructure inspection scenarios.

## 1. Introduction

Crack detection plays a critical role in ensuring the structural integrity and long-term safety of infrastructure systems. As one of the earliest indicators of structural deterioration, timely identification and mitigation of cracks are essential to prevent costly repairs and catastrophic failures [1,2]. Although early-stage cracks may appear insignificant, their propagation can lead to severe degradation and safety hazards. In public infrastructure such as tunnels, bridges, and roadways, cracks can result in substantial human and economic losses while undermining public confidence [3]. Accordingly, early detection and systematic management of cracks remain central to structural health monitoring.

Traditionally, crack inspection has relied on manual visual assessment by trained inspectors. However, this approach is inherently subjective, with outcomes varying based on the inspector’s expertise and environmental conditions such as lighting and viewing angle. Furthermore, manual inspections are labor-intensive and inefficient, particularly for large-scale infrastructure requiring repeated and comprehensive evaluations [4,5]. To address these limitations, image-based crack detection techniques have been introduced. Early methods utilized classical image processing techniques such as edge detection and morphological filtering. For instance, Nnolim [6] proposed an adaptive threshold-based Canny edge detector, while Yun et al. [7] used morphological operations for road crack detection. Parrany and Mirzaei [8] applied adaptive histogram equalization to enhance crack visibility. While effective in simple environments, these methods often fail under complex backgrounds or varying illumination.

To overcome these shortcomings, deep learning-based crack detection models have emerged. Chen et al. [9] combined a transformer and U-Net architectures to handle diverse crack patterns, while Acikgoz et al. [10] introduced multi-scale and residual feature designs for railway crack inspection. Sohaib et al. [11] adopted ensemble learning to improve robustness. A notable example is DeepCrack by Liu et al. [12], which achieved high segmentation accuracy through multi-scale feature fusion. Recent innovations in deep learning have introduced transformer-based models and general-purpose frameworks to further enhance robustness and generalization. For example, DCUFormer [13] and HrSegNet [14] effectively maintain high-resolution contextual information and accurately capture fine-grained crack features. Vision foundation models like Segment Any Crack (SAC) [15] and FlexiCrackNet [16] have achieved few-shot or even zero-shot segmentation by leveraging selective layer adaptation and attention modulation. These architectures consistently perform well on public benchmarks such as Crack500 and CrackForest, often achieving F1-scores above 0.89 under dry or clean conditions. In parallel, techniques such as skeleton-based detection [17], shape-aware feature learning [18], and edge-aware decoding [19] have been proposed to improve performance on faint or discontinuous cracks. However, most existing studies have focused on relatively clean and controlled datasets, and their performance tends to degrade significantly in real-world environments characterized by surface moisture, reflection, and contamination.

To mitigate these real-world limitations, recent research has explored preprocessing techniques to enhance input image quality prior to model inference. For instance, Li et al. [20] integrated contrast limited adaptive histogram equalization (CLAHE) with a YOLO-based model for low-light detection, while Zhang et al. [21] demonstrated that targeted preprocessing significantly improves segmentation accuracy using lightweight models like MobileNetV3. Choi et al. [22] confirmed that improving surface quality directly boosts detection reliability. These studies have advanced beyond simple image enhancement by developing preprocessing strategies tailored to specific environmental conditions. Nonetheless, wet cracks continue to pose persistent challenges. Moisture-induced reflection, staining, and irregular surface textures frequently introduce substantial visual noise, which not only degrade segmentation quality but also obscure early warning signs of damage. In this context, robust preprocessing becomes critical [23,24,25,26].

In parallel with input-level enhancement, several recent works have proposed model-level solutions to address the robustness of crack segmentation in noisy and imperfectly labeled scenarios. Notably, works such as SelectSeg [27] and pixel-accurate crack detection under inaccurate annotations [28] propose uncertainty-based selective learning and robust optimization techniques to improve performance when data is limited or labels are noisy. These methods focus on improving the learning process itself by incorporating noise awareness into the training phase. While effective, such approaches often require complex training pipelines, architecture-specific modifications, or computationally expensive uncertainty estimation.

In contrast, this study explores a complementary yet simpler direction—enhancing input quality through a structured preprocessing pipeline. The key insight is that cleaner and better-contrasted input images can significantly improve segmentation performance even without altering the underlying model architecture. Our proposed method is model-agnostic and applicable to any segmentation network. It consists of structured preprocessing stages, including adaptive thresholding to address local lighting variation, morphological closing and connected component analysis (CCA) to restore crack continuity and remove noise-like fragments, followed by CLAHE and histogram stretching to enhance both local and global contrast. A background fusion is then applied to preserve surface textures while emphasizing crack regions.

This preprocessing pipeline significantly improves the visibility and structural clarity of crack features, providing deep learning models with more informative input representations—especially under noisy, wet environments. To validate its effectiveness, we integrate it with DeepCrack and evaluate performance under dry, wet, and noise-augmented scenarios. The main contributions of this study are as follows:

1. A novel preprocessing scheme that combines morphological closing and CCA is proposed to restore structural continuity in fragmented crack patterns and suppress micro-level noise, especially prevalent in noisy, low-contrast pavement images.
2. To address challenges posed by low visibility and reflective interference in moist environments, the study incorporates CLAHE and histogram stretching techniques. These methods significantly improve the visual distinction between crack and non-crack regions, leading to enhanced feature representation and improved convergence stability during training.
3. A unified and modular preprocessing-to-segmentation pipeline is developed, integrating the above techniques with a deep learning-based segmentation network. This end-to-end system demonstrates superior performance in both accuracy and robustness when applied to wet-surface crack datasets, validating its effectiveness for real-world infrastructure inspection under adverse conditions.

## 2. Methods

**Figure 1:**
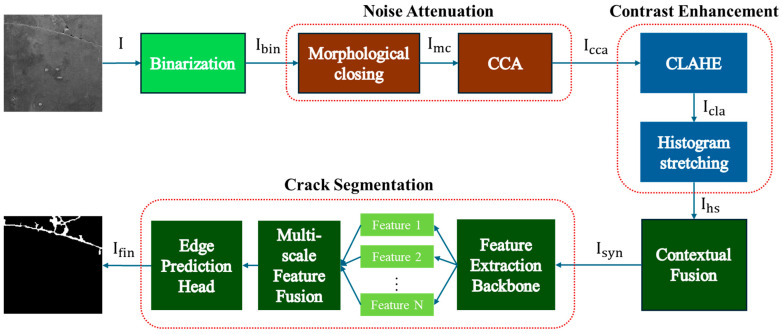
Block diagram of the proposed crack segmentation model.

Detecting wet cracks is particularly challenging due to low contrast and blurred boundaries resulting from surface moisture and staining. To address these issues, this paper proposes a structured preprocessing strategy that enhances crack features and improves segmentation accuracy. The pro-posed pipeline consists of three main stages: binarization, noise attenuation, and contrast enhancement. An overview of the entire pipeline is illustrated in Figure 1.

In the first stage, adaptive thresholding is applied to generate a binary image that separates cracks from the background, ensuring robustness against lighting variations and surface irregularities. In the second stage, morphological closing is used to reconnect fragmented crack segments and fill small gaps. Subsequently, CCA removes small noise-like objects to enhance structural consistency. In the final stage, local contrast is improved using CLAHE, while global contrast is enhanced through histogram stretching, resulting in a sharper image with clearer crack boundaries.

The enhanced crack features are then fused with the original image to form a composite that retains background texture while emphasizing cracks. This fusion allows the segmentation model to learn both structural details of the cracks and contextual environmental information. Unlike conventional mask-based preprocessing, the proposed method preserves visual continuity and realism, which in turn improves the model’s generalization capability. As a result, the final input to the segmentation model contains more salient crack features, leading to improved learning stability and inference accuracy.

### 2.1. Binarization

**Figure 2:**
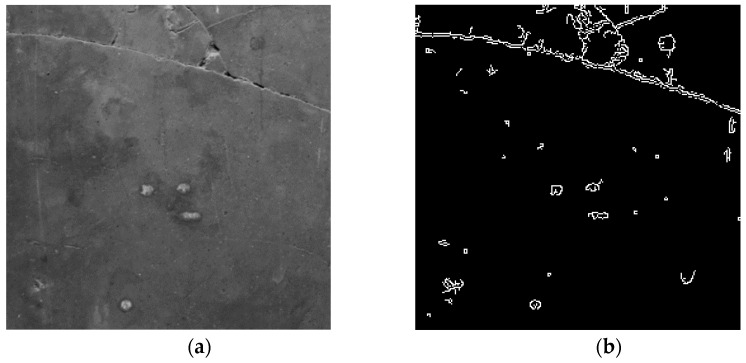
Binarization result using adaptive thresholding: (**a**) original input image; (**b**) binary image after thresholding.

Wet cracks typically exhibit low contrast and blurred edges due to residual moisture and surface staining, making them difficult to distinguish from the background using conventional thresholding techniques. To overcome this limitation, this study employs adaptive thresholding [29], which computes a pixel-wise threshold based on the local intensity distribution. Specifically, the threshold for each pixel is determined by the mean intensity of its surrounding region, which enhances robustness to lighting variations. The adaptive thresholding function is defined as

(1) $$I_{bin}\left(x,y\right)=\left\{\begin{array}{l} 1,\;if\;I\left(x,y\right)>\mu \left(x,y\right)-C\\ 0,\;otherwise \end{array},\right.$$

where I(x,y) denotes the original image intensity at a pixel (x,y), μ(x,y) represents the Gaussian-weighted local mean, and C is a user-defined constant that controls the threshold offset. This approach effectively distinguishes cracks from the background, reducing the likelihood of missing faint or partially illuminated cracks. Figure 2 illustrates the binarization result using adaptive thresholding, where crack regions are more clearly segmented compared to the original input image.

### 2.2. Noise Attenuation

Even after binarization, residual noise often remains in the image. In the case of wet cracks, residual moisture, dust particles, and surface reflections can introduce non-crack artifacts that adversely affect segmentation performance. To mitigate this, a sequential application of morphological closing and CCA is proposed to suppress noise while preserving crack continuity.

Morphological closing [30] is used to smooth object boundaries in binary images and eliminate small holes or background noise within crack regions, which reinforces structural consistency. It consists of a dilation followed by an erosion and is formally defined as:

(2) $$I_{mc}=\left(I_{bin}⊕F\right)⊖F$$

where F denotes a predefined m×m structuring element, ⊕ represents the dilation operation, and ⊖ denotes erosion. During dilation, white (foreground) regions are expanded by the influence of neighboring white pixels, which reconnects broken crack segments. The subsequent erosion restores the shapes of these expanded regions while removing artifacts introduced during dilation. This operation is particularly effective in reducing crack fragmentation and discontinuity caused by surface stains or reflections in moist environments.

**Figure 3:**
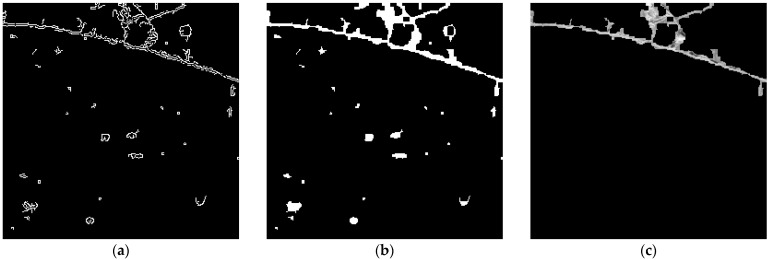
Noise suppression results: (**a**) binarized image before noise suppression; (**b**) after morphological closing; (**c**) after morphological closing and CCA.

Following the morphological closing operation, CCA is applied to eliminate small, irrelevant objects. CCA identifies connected pixel regions in a binary image and removes those whose area falls below a predefined threshold. In environments with wet cracks, surface contaminants such as dust, water droplets, and reflections often generate artifacts that resemble crack patterns. By evaluating the area of each connected region, CCA-based filtering effectively removes such noise elements. The CCA-based noise suppression is defined as

(3) $$I_{cca}\left(x,y\right)=\left\{\begin{array}{l} I_{mc}(x,y),\;if\;A(i)\geq A_{bin}\\ 0,\;otherwise \end{array},\right.$$

where A(i) denotes the area of the *i*th connected component and $A_{bin}$ is the minimum area threshold. Components smaller than this threshold are unlikely to represent true cracks and are thus excluded to enhance detection reliability. Figure 3 shows a comparison of the results before and after noise attenuation. The binarized image contains numerous small, dot-like noise artifacts. After applying morphological closing and CCA sequentially, the crack regions become more prominent while background noise is substantially reduced.

### 2.3. Contrast Enhancement

Despite the preceding noise attenuation steps, one of the major challenges in crack detection remains the low contrast between cracks and the background. This issue is particularly prominent in wet crack environments, where internal moisture and surface reflections can cause cracks to visually blend into the surrounding texture. To address this, a sequential contrast enhancement process is applied, consisting of CLAHE followed by histogram stretching. This process improves the visual distinction of cracks, which in turn enhances segmentation performance.

**Figure 4:**
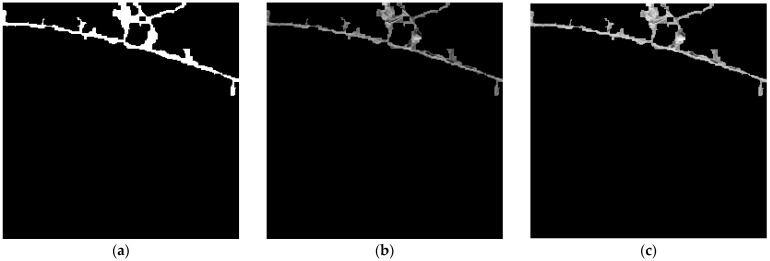
Contrast enhancement results: (**a**) original image before enhancement; (**b**) after applying CLAHE; (**c**) after applying CLAHE and histogram stretching.

CLAHE [31] enhances local contrast by dividing the image $I_{cca}\left(x,y\right)$ into a grid of contextual subregions $T_{i,j}$ rather than applying a global transformation. For each subregion, a histogram is computed, and its cumulative distribution function (CDF) is used to redistribute pixel intensities. A clip limit is applied to each histogram to prevent over-amplification of frequently occurring brightness levels, with the excess redistributed across the range of intensity values [32]. The contrast-enhanced image using CLAHE is expressed as

(4) $$I_{cla}\left(x,y\right)=\left(\gamma -1\right)\times CDF_{i,j}\left(I_{cca}\left(x,y\right)\right),$$

where $CDF_{i,j}$ denotes the CDF for block $T_{i,j}$, and γ is the clip limit. CLAHE is particularly effective in amplifying crack visibility within local regions while preventing contrast oversaturation in relatively uniform background areas. However, to further enhance the global distinction between crack and background intensities, histogram stretching is applied after CLAHE. This linear transformation expands the dynamic range of the image according to

(5) $$I_{hs}\left(x,y\right)=\frac{I_{cla}\left(x,y\right)-I_{min}}{I_{max}-I_{min}}\times \left(L-1\right),$$

where $I_{min}$ and $I_{max}$ denote the minimum and maximum intensity values in the image, and L is the maximum displayable intensity level. This step further accentuates the brightness difference between cracks and the background, producing a sharper, more distinguishable image. Figure 4 illustrates the results of contrast enhancement using CLAHE followed by histogram stretching. In the original image, cracks and the background exhibit similar intensity levels, making separation difficult. After enhancement, the crack contours become significantly more distinct and visually pronounced. This improvement not only facilitates clearer visual interpretation but also enables the segmentation model to learn crack features and patterns more effectively.

### 2.4. Crack Segmentation

**Figure 5:**
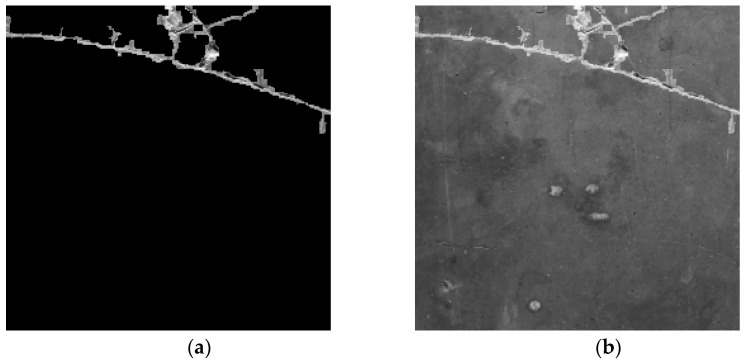
Synthesized image results: (**a**) before background fusion; (**b**) after background fusion. The fused image emphasizes crack regions while preserving the original background, enhancing both interpretability and segmentation performance.

The final step of the proposed framework involves crack segmentation using a deep learning model. This is performed on a synthesized image generated by merging the contrast-enhanced image $I_{hs}\left(x,y\right)$ with the original image $I\left(x,y\right)$. The result is a composite image $I_{syn}\left(x,y\right)$ that preserves both the enhanced crack features and the contextual background. This fusion allows the model to benefit from salient crack representations while maintaining the spatial realism of the input. The fusion operation is defined as

(6) $$I_{syn}\left(x,y\right)=D\times I_{hs}\left(x,y\right)+\left(1-I_{hs}\left(x,y\right)\right)\times I\left(x,y\right),$$

where D is a constant that amplifies the intensity of crack regions. The fusion ensures that visually salient crack features are emphasized without distorting background textures. Figure 5 illustrates the result of this background fusion process, where the crack regions become more distinguishable compared to the original image, and small or faint cracks are enhanced without losing spatial context. In the fused image, pixels identified as cracks are retained as white (255), while non-crack pixels are replaced with corresponding grayscale values from the original image. Compared to Figure 5a, where small cracks are not visible due to brightness variation and noise, Figure 5b highlights fine cracks more clearly, demonstrating the effectiveness of the proposed preprocessing method. This step improves the visual clarity of crack regions while maintaining background details, which supports more intuitive and reliable crack detection in real-world scenarios.

The composite image $I_{syn}\left(x,y\right)$ is then passed to a deep learning-based segmentation network, DeepCrack [12]. DeepCrack is a convolutional neural network (CNN) designed for crack segmentation using a multi-scale feature fusion architecture. It effectively captures crack patterns of varying shapes, widths, and orientations, making it suitable for both fine and prominent cracks in complex environments. DeepCrack adopts an encoder–decoder structure comprising five convolutional stages with 3 × 3 kernels. Each encoder stage consists of convolutional layers followed by ReLU activation and max pooling operations, enabling the model to progressively reduce spatial dimensions while capturing deeper semantic features. In the decoder, transposed convolutional layers are used for upsampling, and skip connections between corresponding encoder and decoder layers help retain spatial information and boundary precision. In addition, multi-scale features extracted at different depths are aggregated through a feature fusion module, allowing the model to capture both fine-grained crack textures and broader contextual patterns. The final segmentation output is generated by a 1 × 1 convolution followed by a sigmoid activation, producing a binary mask that distinguishes crack pixels from the background. This architecture is particularly effective in preserving crack continuity and detecting faint or fragmented cracks across variable scales and orientations.

To optimize segmentation accuracy, the model is trained using a composite loss function that combines Binary Cross Entropy (BCE) loss, ${ℒ}_{BCE}$, and Dice loss, ${ℒ}_{Dice}$, to balance pixel-level classification and mask-level overlap. The total loss function is defined as:

(7) $${ℒ}_{total}=\lambda_{1}{ℒ}_{BCE}+\lambda_{2}{ℒ}_{Dice},$$

where $\lambda_{1}$ and $\lambda_{2}$ are weighting coefficients. The BCE loss measures the pixel-wise classification error between the predicted probability map (PM) and the ground truth (GT), and is given by

(8) $${ℒ}_{BCE}=-\frac{1}{N}\sum_{i=1}^{N}[GT_{i}\times \log\left(PM_{i}\right)+(1-GT_{i})\times log(1-PM_{i})],$$

where N is the total number of pixels, $GT_{i}$ is the ground truth value of pixel i, and $PM_{i}$ is the predicted mask value. The Dice loss measures the overlap between the predicted mask and the ground truth, and is defined as

(9) $${ℒ}_{Dice}=1-\frac{2\sum_{i=1}^{N}GT_{i}\times PM_{i}+\varepsilon }{\sum_{i=1}^{N}GT_{i}+\sum_{i=1}^{N}PM_{i}+\varepsilon },$$

where ε is a small constant to prevent division by zero. The combined use of BCE and Dice losses ensures that both the precision and recall of crack segmentation are optimized, particularly in unbalanced datasets where crack pixels are spare compared to background pixels.

The integration of the preprocessing pipeline with the DeepCrack model results in significantly improved segmentation performance, as the input image provides clearer and more distinguishable crack features. This joint approach enables the model to learn robust representations, even under challenging conditions such as low contrast, moisture-induced noise, and irregular crack morphology.

## 3. Experimental Results

This section presents experimental results validating the effectiveness of the proposed preprocessing pipeline and evaluating segmentation performance under various crack conditions. A set of controlled experiments was conducted using multiple datasets, and the proposed model was quantitatively compared with state-of-the-art segmentation approaches of similar architecture.

### 3.1. Experimental Setup

For model training and evaluation, a unified dataset was constructed by combining publicly available dry crack datasets with real-world wet crack images that exhibit challenging visual distortions, such as uneven lighting, surface reflections, residual moisture, and surface staining. The wet crack images were selectively collected from various publicly accessible online repositories and previous studies [18,19], and were used solely for non-commercial academic research. While the dry crack datasets provide favorable learning conditions with clearly defined crack boundaries and high contrast, the wet crack dataset introduces complex backgrounds and realistic distortions, enabling robust evaluation under practical conditions. The final dataset consists of 1772 images with 909 for training, 331 for validation, and 532 for testing. Model training and inference were performed using Python 3.11.9, PyTorch 2.2.2, and CUDA 11.8 on a workstation equipped with an NVIDIA GeForce RTX 3060 GPU (NVIDIA Corp., Santa Clara, CA, USA) and an Intel Core i7-8700 CPU (Intel Corp., Santa Clara, CA, USA). Additionally, the key parameters used in the preprocessing stage were optimized through iterative experiments. The clip limit, γ, for CLAHE was set to 2, which was found to be appropriate for enhancing local contrast of cracks in low-light conditions while preventing noise amplification due to excessive contrast enhancement. For instance, when the clip limit was set too low, cracks were not sufficiently distinguished from the background; conversely, overly high values caused unwanted noise—such as reflections or surface stains—to be excessively emphasized. In the case of CCA, the area threshold was set to 85 pixels. This value was determined to effectively remove small, irrelevant objects such as moisture reflections or surface dust, while preserving fine cracks commonly observed in real-world images.

For the segmentation task, we adopted DeepCrack which features an encoder–decoder structure with five convolutional stages using 3 × 3 kernels. Each encoder stage consists of convolutional layers followed by ReLU activation and max pooling to reduce spatial dimensions while increasing semantic depth. The decoder mirrors this structure, using transposed convolutions for upsampling. Additionally, skip connections between encoder and decoder layers help retain spatial resolution and preserve fine crack boundaries. To further enhance performance, multi-scale feature fusion is performed across different layers, allowing the model to simultaneously capture local texture variations and global structural patterns. The final output is generated by a 1 × 1 convolution followed by a sigmoid activation, producing a binary segmentation mask that distinguishes cracks from the background.

Segmentation performance was assessed using standard quantitative metrics, including the Dice coefficient, Intersection over Union (IoU), Precision, Recall, and F1-Score [12]. These metrics evaluate pixel-level correspondence between the PM and the GT. In particular, the Dice coefficient effectively captures the overlap between predicted and actual crack regions and is robust to class imbalance. It is defined as

(10) $$Dice=\frac{2\sum_{i}PM_{i}\times GT_{i}}{\sum_{i}PM_{i}+\sum_{i}GT_{i}}$$

The IoU metric quantifies the degree of overlap between the PM and the GT, emphasizing the ratio of correctly detected pixels relative to the union of predicted and actual crack regions. It is defined as

(11) $$IoU=\frac{2\sum_{i}PM_{i}\times GT_{i}}{\sum_{i}PM_{i}+\sum_{i}GT_{i}-\sum_{i}PM_{i}\times GT_{i}}$$

Precision is defined as the proportion of correctly predicted crack pixels to all pixels predicted as cracks, calculated as TP/(TP + FP), and Recall represents the ratio of correctly identified crack pixels to all actual crack pixels, calculated as TP/(TP + FN). Here, TP, FP, and FN denote the number of true positives, false positives, and false negatives, respectively. The F1-score is the harmonic mean of Precision and Recall, providing a balanced measure of accuracy that considers both false positives and false negatives. It is defined as

(12) $$F1\;score=\frac{2\times Precision\times Recall}{Precision+Recall}$$

### 3.2. Performance Evaluation

#### 3.2.1. Comparison of Dry Crack Segmentation Performance

To quantitatively evaluate the performance of the proposed model in environments with dry cracks, comparative experiments were conducted against several representative crack detection models: DeepCrack [12], PAN [33], CrackNet [34], SegNet [35], and U-Net [36]. These models are known for their reliable performance under relatively clean and well-contrasted conditions and are based on diverse network architectures and training strategies. The proposed model adopts an encoder–decoder architecture comprising five 2D convolutional stages with 3 × 3 kernels and small receptive fields. In the encoder, each stage is followed by ReLU activations and max pooling layers, while the decoder utilizes transposed convolutions for upsampling. The number of feature channels increases progressively up to 512. Skip connections are employed to link corresponding encoder and decoder stages. Finally, multi-scale features are aggregated, and a 1 × 1 convolutional layer with sigmoid activation produces the final binary segmentation mask.

Table 1 summarizes the segmentation performance across five evaluation metrics: Dice coefficient, IoU, Precision, Recall, and F1-Score. The baseline models generally achieved Dice and F1-scores between 0.7 and 0.8, indicating reasonable segmentation accuracy under normal conditions. In contrast, the proposed model outperformed all baselines across all metrics. Notably, it achieved a Dice coefficient of 0.9671 and an IoU of 0.9342, representing a substantial improvement over existing methods. Both Precision (0.9557) and Recall (0.9714) exceeded 0.95, demonstrating high accuracy and reliability in crack segmentation.

**Figure 6:**
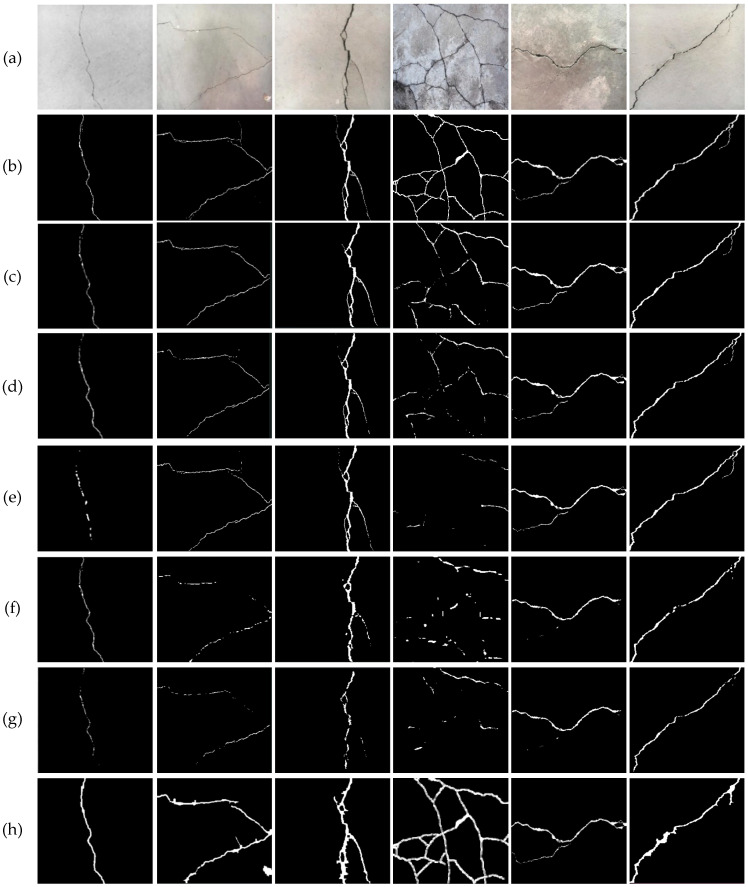
Visual comparison of segmentation results in environments with dry cracks: (**a**) original image; (**b**) ground truth; (**c**) DeepCrack [12]; (**d**) PAN [33]; (**e**) CrackNet [34]; (**f**) SegNet [35]; (**g**) U-Net [36]; (**h**) proposed model.

Figure 6 presents the visual comparison results under dry conditions. Figure 6a shows the original image, while Figure 6c–h correspond to the outputs from DeepCrack, PAN, CrackNet, SegNet, U-Net, and the proposed model, respectively. While the baseline models generally performed well in detecting primary crack regions, they exhibited issues such as unclear boundaries, crack discontinuities, and misclassification of small noise artifacts. In contrast, the proposed model provided clearer segmentation boundaries, maintained continuity even in fine crack regions, and suppressed false positives effectively. These results confirm that the proposed model successfully addresses key limitations of existing methods in environments with dry cracks, including robustness to noise, improved boundary definition, and superior overall segmentation quality.

#### 3.2.2. Comparison of Wet Crack Segmentation Performance

Table 2 presents a quantitative comparison of crack segmentation performance under wet conditions. Existing models, including DeepCrack, PAN, CrackNet, SegNet, and U-Net, exhibited poor performance, with Dice coefficients around 0.2 and IoU values between 0.10 and 0.17. Precision and Recall scores were also below 0.5, indicating limited reliability. This degradation is primarily due to reduced contrast and visual distortions caused by surface moisture and reflections, which hinder accurate crack detection. In contrast, the proposed model demonstrated significantly superior performance across all evaluation metrics, achieving values above 94%. The Dice coefficient improved by over 0.6 compared to the baseline models, clearly demonstrating the effectiveness of the proposed preprocessing strategy in enhancing segmentation performance under adverse visual conditions.

**Figure 7:**
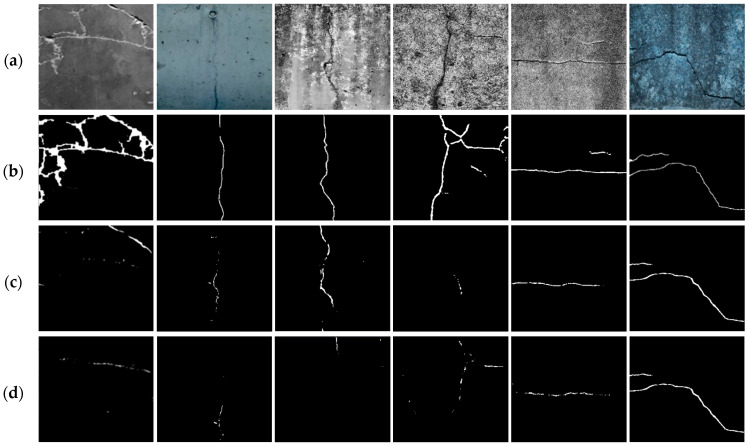

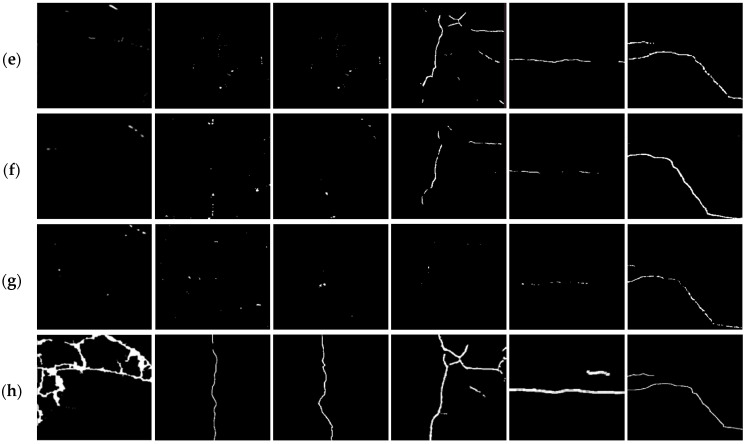
Visual comparison of segmentation results in environments with wet cracks: (**a**) original image; (**b**) ground truth; (**c**) DeepCrack [12]; (**d**) PAN [33]; (**e**) CrackNet [34]; (**f**) SegNet [35]; (**g**) U-Net [36]; (**h**) proposed model.

The superior segmentation capability of the proposed model is further demonstrated through visual comparisons. Figure 7 presents the segmentation results of various models in environments with wet cracks. While existing models exhibit incomplete crack separation and blurred boundaries due to moisture-induced distortions, the proposed model effectively delineates crack contours and preserves their overall shape and continuity. These visual results, in conjunction with the quantitative metrics, highlight the proposed model’s robustness in handling low-contrast and noise-prone conditions. The findings confirm that the proposed method addresses key limitations of conventional models under wet crack conditions and offers substantial improvements for real-world crack detection systems.

#### 3.2.3. Comparison of Segmentation Performance in Noisy Environments

To further evaluate robustness of the proposed model, Gaussian noise was deliberately introduced into the wet crack dataset to simulate challenging real-world noisy conditions. Table 3 presents a quantitative comparison of segmentation performance under these noisy environments. Most baseline models, including PAN, CrackNet, and SegNet, exhibited a substantial drop in performance, with Dice coefficients and IoU values falling below 0.2. This significant decline highlights their susceptibility to background interference and boundary uncertainty caused by noise. In particular, models such as PAN, CrackNet, and SegNet showed drastic decrease in Dice coefficients, underscoring their limited noise robustness.

In contrast, the proposed model maintained significantly higher segmentation accuracy, achieving a Dice coefficient of 0.8461, IoU of 0.7370, Precision of 0.8596, Recall of 0.8333, and F1-score of 0.8462. These results clearly demonstrate the model’s strong generalization and representational capability, enabling accurate segmentation of crack regions despite the presence of substantial visual noise. The balanced precision and recall values further confirm its stability and robustness.

This enhanced noise robustness is primarily attributed to the proposed preprocessing pipeline, which effectively suppresses noise while preserving crack continuity. The adaptive thresholding stage adapts to local intensity variations, improving crack discrimination even in low-contrast, noisy regions. Morphological closing reconnects fragmented crack segments, reducing the effects of noise-induced discontinuities. CCA filters out small noise-line artifacts that do not meet size criteria, effectively removing irrelevant background disturbances such as moisture reflections and dust particles.

Furthermore, the contrast enhancement steps, including CLAHE and histogram stretching, increase both local and global contrast, which sharpens crack boundaries and improves the distinction between cracks and noisy backgrounds. The fusion of the enhanced image with the original preserves background texture while emphasizing crack features, providing the segmentation network with richer and more reliable inputs.

Overall, these preprocessing strategies significantly contribute to the model’s resilience against noise, enabling robust crack segmentation in noisy wet environments. Such robustness is essential for practical applications, where imaging conditions are often uncontrolled and affected by environmental factors.

#### 3.2.4. Justification of Experimental Design

While the proposed method is fundamentally a preprocessing strategy and can, in principle, be applied to various segmentation models, this study focuses on evaluating its effectiveness within an integrated pipeline using DeepCrack as the backbone network. DeepCrack was chosen due to its demonstrated effectiveness in crack detection and its incorporation of multi-scale feature learning, which enables it to benefit substantially from improved input quality.

The primary objective of this experiment was to assess whether the proposed preprocessing pipeline can meaningfully improve segmentation performance under real-world adverse conditions—particularly in the presence of wet cracks and visual noise. The significant performance gains observed in these scenarios, especially in terms of Dice coefficient, IoU, and F1-score, provide strong evidence of the method’s robustness and practical applicability. While integrating the preprocessing pipeline with multiple segmentation architectures would offer deeper insights into its model-agnostic capabilities, the current implementation serves as a proof of concept. It clearly demonstrates that the proposed strategy can deliver substantial improvements in a realistic and challenging detection context. Future work will explore applying the method across diverse segmentation frameworks to further validate its generalizability.

## 4. Discussion

The primary contribution of this study lies in demonstrating that a well-designed, modular preprocessing pipeline can significantly enhance crack segmentation performance in visually challenging environments without modifying the architecture of the segmentation model itself. By systematically addressing issues such as visual noise, low contrast, and crack discontinuities, the proposed method provides a robust enhancement to the input data, thereby improving the effectiveness of standard deep learning segmentation models. While DeepCrack was adopted as the backbone in this study, the modular design of the preprocessing framework ensures compatibility with various segmentation architectures, supporting model-agnostic integration.

The observed performance improvements can be attributed to several key components within the pipeline. Adaptive thresholding enables robust crack localization under uneven lighting conditions. Morphological closing and CCA facilitate the removal of small, irrelevant noise artifacts while preserving crack continuity. Furthermore, the combination of CLAHE and histogram stretching enhances both local and global contrast, sharpening the visibility of fine cracks. Finally, image fusion between the enhanced and original images preserves background realism while highlighting crack regions, resulting in more informative inputs for the segmentation model.

Although comparisons in this study focused primarily on classical and widely used models such as U-Net, SegNet, and PAN, these baselines remain representative of practical applications in infrastructure inspection. However, we acknowledge that several recent works—such as CrackFormer [37], RHACrackNet [38], OUR-Net [39], and AutoCrackNet [40]—have introduced advanced architectures tailored specifically for pavement crack detection. These models integrate mechanisms such as multi-scale transformers, residual attention, and self-calibrated convolutions to achieve high performance in ideal conditions. While a direct experimental comparison with these cutting-edge methods was beyond the scope of this study, the proposed preprocessing pipeline is inherently compatible with such architectures. As a result, the pipeline could serve as a complementary input enhancement module that may further boost the accuracy and robustness of modern segmentation networks, especially in low-contrast and noisy environments. This integration will be explored in future work.

Despite its demonstrated advantages, the proposed method has several limitations that should be addressed in future work. First, several preprocessing parameters—such as the CLAHE clip limit and CCA area threshold—were empirically determined based on iterative testing. While these settings were effective for the current dataset, their generalization to other datasets or imaging conditions may require additional tuning. Incorporating data-driven or adaptive parameter selection strategies could improve robustness across diverse scenarios. Second, the current evaluation was primarily conducted on pavement-type surfaces under wet and noisy conditions. As such, the applicability of the proposed pipeline to other structural materials remains to be fully validated. Future studies should expand the dataset to encompass a broader range of infrastructure types and material characteristics. Third, although the preprocessing pipeline is designed to be model-agnostic, the experimental validation was limited to a single segmentation network. Benchmarking the pipeline with various architectures, including lightweight real-time models and recent transformer-based frameworks, would offer further insight into its generalizability and versatility across both traditional and emerging segmentation paradigms. Finally, the current implementation is not optimized for real-time deployment. The multiple sequential processing steps may introduce latency, limiting the method’s suitability for time-sensitive applications such as mobile or robotic inspection systems. To this end, optimizing the pipeline for computational efficiency—through parallelization, GPU acceleration, or algorithmic simplification—will be crucial to supporting real-time infrastructure monitoring.

In summary, the proposed preprocessing strategy presents a scalable and effective solution to a long-standing challenge in crack detection: achieving high segmentation accuracy in low-visibility and noise-prone conditions. While the current study demonstrates its utility using conventional segmentation models, the pipeline holds strong potential for integration with cutting-edge architectures, further enhancing its relevance to the state-of-the-art. Future research will focus on extending its applicability across infrastructure domains, improving efficiency for real-time deployment, and exploring its synergy with the latest deep learning models for structural health monitoring.

## 5. Conclusions

This study proposed a preprocessing-based framework to enhance crack segmentation performance, particularly in visually challenging environments characterized by surface moisture, low contrast, reflections, and noise. The proposed pipeline systematically addresses the core challenges of wet crack detection through a sequence of structured stages: adaptive thresholding to handle local illumination variations; morphological closing and CCA to suppress noise while preserving crack continuity; and a contrast enhancement strategy combining CLAHE and histogram stretching to improve both local and global visibility of cracks. Additionally, a background fusion technique emphasizes crack regions while retaining the original image texture, providing the segmentation model with both structural clarity and contextual realism.

To validate its effectiveness, extensive experiments were conducted under three distinct conditions: dry, wet, and noisy settings. The proposed method consistently outperformed existing state-of-the art models across all test scenarios, achieving a Dice coefficient of 0.9698 and an F1-score of 0.9702 in wet conditions—a dramatic improvement over baseline methods whose performance dropped below 0.3. Furthermore, even exposed to synthetic noise, the model maintained a high Dice coefficient of 0.8461, demonstrating strong resilience and generalization capability.

While this study utilized DeepCrack as the base segmentation network, the preprocessing framework is designed to be model-agnostic and can be integrated with a wide range of segmentation architectures. Future work will explore its application to various infrastructure types, such as bridges and tunnels, and diverse material surfaces including concrete, asphalt, and metal. In addition, efforts will be directed toward optimizing the pipeline for real-time operation and extending it to support downstream tasks such as crack classification, progression tracking, and structural health assessment. These advancements aim to facilitate the development of comprehensive, automated infrastructure monitoring systems suitable for deployment in real-world environments.

## Figures and Tables

**Table 1:** Comparison of dry crack segmentation performance.

Segmentation Model	Dice Coefficient	IoU	Precision	Recall	F1-Score
DeepCrack [12]	0.7293	0.5834	0.8580	0.6484	0.7381
PAN [33]	0.7958	0.6650	0.8535	0.7492	0.7979
CrackNet [34]	0.7916	0.6594	0.8539	0.7419	0.7838
SegNet [35]	0.7493	0.6064	0.8060	0.7072	0.7528
U-Net [36]	0.8018	0.6727	0.8531	0.7592	0.8034
Proposed model	0.9671	0.9342	0.9557	0.9714	0.9657

**Table 2:** Comparison of wet crack segmentation performance.

Segmentation Model	Dice Coefficient	IoU	Precision	Recall	F1-Score
DeepCrack [12]	0.2150	0.1430	0.5120	0.1590	0.2300
PAN [33]	0.2493	0.1567	0.4697	0.2012	0.2807
CrackNet [34]	0.2418	0.1484	0.4982	0.1839	0.2669
SegNet [35]	0.2491	0.1759	0.4874	0.1888	0.2691
U-Net [36]	0.2014	0.1325	0.5416	0.3567	0.2912
Proposed model	0.9698	0.9422	0.9755	0.9632	0.9702

**Table 3:** Comparison of wet crack segmentation performance in noisy environments.

Segmentation Model	Dice Coefficient	IoU	Precision	Recall	F1-Score
DeepCrack [12]	0.1891	0.1274	0.4529	0.1371	0.1983
PAN [33]	0.0503	0.0271	0.3872	0.0292	0.0540
CrackNet [34]	0.0318	0.0581	0.3922	0.1322	0.0602
SegNet [35]	0.0482	0.0326	0.2864	0.0623	0.0595
U-Net [36]	0.1644	0.0992	0.5165	0.1101	0.1794
Proposed model	0.8461	0.7370	0.8596	0.8333	0.8462

## Data Availability

The data that support the findings of this study are not available due to institutional policy.
